# Proteinaceous Venom Expression of the Yellow Meadow Ant, *Lasius flavus* (Hymenoptera: Formicidae)

**DOI:** 10.3390/toxins15020106

**Published:** 2023-01-26

**Authors:** Binwei Wang, Qiaoli Xiao, Xun Li, Jun Wang, Jiaying Zhu

**Affiliations:** 1 Key Laboratory of Forest Disaster Warning and Control of Yunnan Province, College of Biodiversity Conservation, Southwest Forestry University, Kunming 650224, China; 2 Key Laboratory for Forest Resources Conservation and Utilization in the Southwest Mountains of China, Ministry of Education, College of Biodiversity Conservation, Southwest Forestry University, Kunming 650224, China

**Keywords:** venom, ant, transcriptome, *Lasius flavus*, Hymenoptera

## Abstract

Ants are one of the important groups of venomous animals with about 14,000 described species. Studies so far focused on the discovery of venom proteins are only available for limited stinging ants, and the proteinaceous compositions of the stingless ants are completely unknown. Here, we used the transcriptomic approach to identify venom components from the yellow meadow ant, *Lasius flavus*, a stingless ant. The transcriptomic analysis yielded an extraordinary simplicity of the venom expression profile, with 17 venom proteins, such as phospholipase B, odorant binding protein, and apolipoprotein D. Ten of them were discovered as novel toxins for future functional investigations. Quantitative real time PCR analysis revealed that genes encoding the identified venom proteins display exclusively or highly expression profiles in venom glands, validating them as venom compositions. Our findings contribute to the understanding of the evolutional diversity of toxins between stinging and stingless ants.

## 1. Introduction

Ants (Hymenoptera: Formicidae) are one of the most numerically abundant groups of venomous animals, with approximately 14,000 known species and an estimated 11,000 additional species awaiting description [1]. The great diversity of extant ant species is classified into 17 subfamilies, which evolved from the common ancestor of modern ants around 145 million years ago [2,3]. The venom of ants derived from venom glands can be used in predating, defense, or offense, but also serve as chemical communication [4,5]. Ant venom is composed of a mixture of peptides, proteins, and other chemicals, such as formic acid, biogenic amines, and alkaloids, exhibiting diverse activities, such as insecticidal, antimicrobial, and antinociceptive activities [6,7]. It is a promising resource of novel bioactive molecules that can be used for biopesticides and drug development [8].

Until recently, alkaloids in the venom of ants have been relatively well-studied [9], while other venomous chemical compounds and proteinaceous venoms remain largely unveiled. Proteinaceous venom components have only been deciphered in a limited number of ants from several subfamilies including Ectatomminae, Myrmeciinae, Myrmicinae, Ponerinae, Paraponerinae, and Pseudomyrmecinae, mostly focused on emblematic species [10,11,12,13,14,15,16,17]. The characterization of venom components from more ant species belonging to diverse subfamilies will contribute to a better understanding of the evolution of ant venoms and facilitate their applications.

The Formicinae with more than 3,000 described species distributed among 51 genera are the second-largest subfamily of ants [2]. The proteinaceous venoms of this subfamily have not received attention. The yellow meadow ant, *Lasius flavus*, is a species from this group, which is known for creating anthills in grassland and downland habitats, and is commonly found in Asia, Central Europe, and Northern Africa [18,19]. In this study, we applied transcriptomics to reveal the venom expression of the yellow meadow ant.

## 2. Results

### 2.1. Overview of Transcriptome

**Figure 1:**
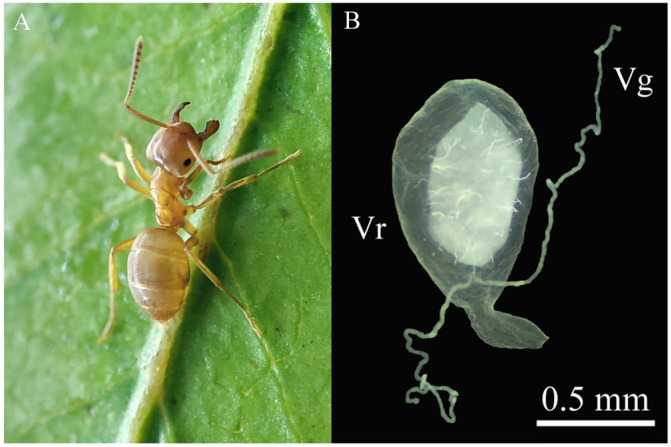
Morphology of *Lasius flavus* (**A**) and its venom apparatus (**B**). Vg, venom gland; Vr, venom reservoir.

In order to construct a transcriptomic database for revealing the venom profile of *L. flavus*, cDNA libraries were built with total RNA extracted from venom the gland and carcass, the body deprived of a venom apparatus. The venom apparatus of this ant was shown in Figure 1. They were deeply sequenced with the Illumina paired end reads sequencing. A total of 41,335,556 raw reads were generated, which were reduced to 40,450,976 clean reads after removing the adaptor and low-quality reads (Table 1). The Q20 and Q30 scores were more than 92% in each library, respectively. The clean reads were pooled for de novo assembly with Trinity software and assembled into 51,015 unigenes, with their average length of 895.17 bp and N50 value of 263 bp. From the unigenes, 42,047 coding sequences were predicted with Transdecoder. After BLAST similarity searches against the National Center for Biotechnology Information (NCBI) non-redundant (Nr) Insecta database, a total of 24,721 unigenes were annotated. Among these sequences, a total of 7947 unigenes (15.58%) had significant homology against *L. niger*, followed by those against *Heliconius pachinus* (3362, 6.59%), *Formica selysi* (1933, 3.79%), and *Cataglyphis niger* (1700, 3.33%) (Figure S1). In total, 17,261 unique unigenes were annotated in Gene Ontology (GO), of which 6821, 7323, and 7689 unigenes were respectively classified into the molecular function, cellular component, and biological process (Figure S2).

### 2.2. Venom Expression Profile Revealed by Transcriptomics

Based on a transcriptomic approach, 17 venom components were identified from *L. flavus* (Table 2 and Table S1). Of them, seven venom proteins were annotated as phospholipase B, odorant binding protein, apolipoprotein D, takeout protein, carbonic anhydrase, nose resistant to fluoxetine protein, and thioredoxin reductase. The other ten have no similarity with protein sequences deposited in the Nr database, which are novel venom toxins. Interestingly, seven of them (UN4-UN10) are peptidic toxins with the length of the amino acid sequence below 100 aa.

### 2.3. Expression Pattern of Venom Gene

**Figure 2:**
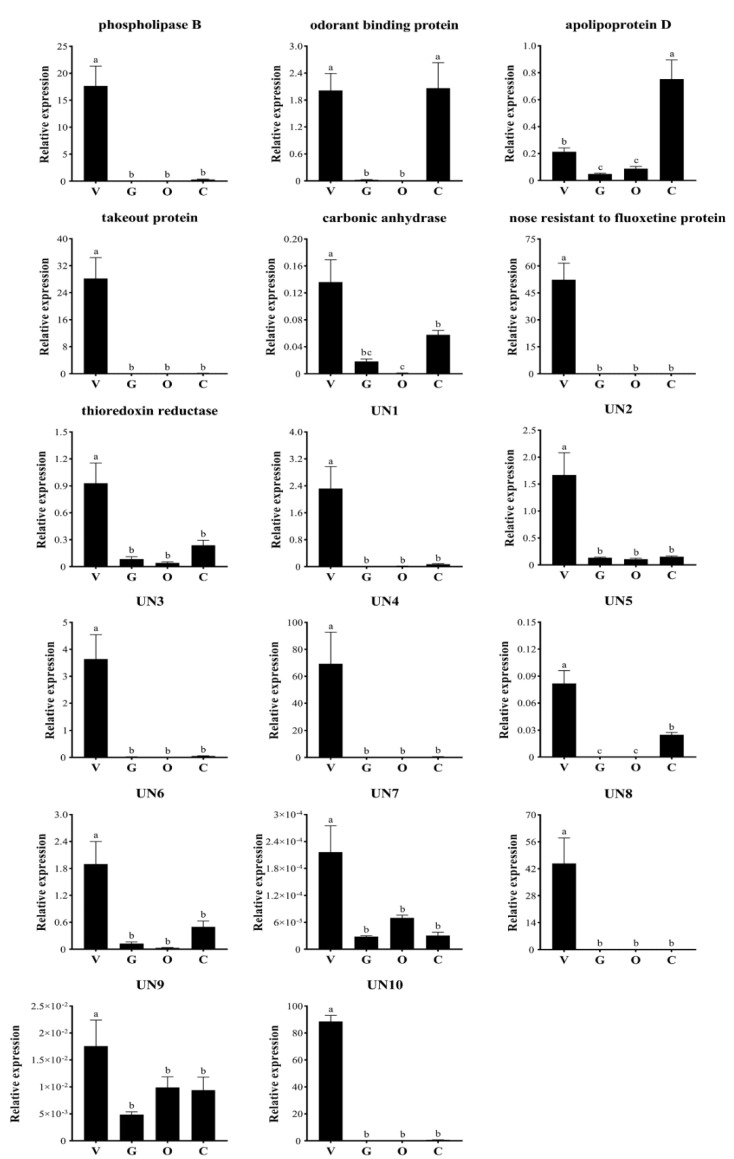
Expression profiles of venom genes in different tissues of *Lasius flavus* workers. V, venom gland; G, gut; O, ovary; C, carcass (body deprived of venom apparatus, gut, and ovary). All values in the figure are represented as mean ± standard deviation. Bars labeled with different letters (a–c) are significantly different.

The expression profiles of all identified putative venom genes were determined in different tissues of *L. flavus* workers using quantitative real time PCR (qPCR) (Figure 2). The results indicated that most of them show abundant expression patterns in the venom gland. Eight venom genes, including phospholipase B, takeout protein, nose resistant to fluoxetine protein, UN1, UN3–4, UN8, and UN10, are specifically expressed in the venom gland. The transcriptional levels of carbonic anhydrase, thioredoxin reductase, UN2, UN5–7, and UN9 in the venom gland are significantly higher than in the gut, ovary, and carcass. Although the gene expressions of odorant binding protein and apolipoprotein D in the venom gland are comparable to or lower than in the carcass, their transcriptional levels in the venom gland are significantly higher than in the gut and ovary. Overall, qPCR results validate that the above 17 proteins identified via the transcriptomic approach are venom components of *L. flavus*.

## 3. Discussion

The venom composition of ants has received less attention with limited publication to date [10,11,12,13,14]. These previously published studies revealed the large diversity of venom components from each species based on the transcriptomic, proteomic, or proteo-transcriptomic approach [10,13,20,21]. For instance, 92 toxin-like peptides and proteins were identified from *Odontomachus monticola*, a predatory ant species in the subfamily Ponerinae [10]. These species studied have a stinger associated with a venom apparatus used to inject venom for defending against predators and hunting prey [5]. The yellow meadow ant, *L. flavus*, used in this study lacks the stinger and does not employ venom to overcome prey. Only 17 venom proteins were profiled from this Formicinae species. In contrast to rich venom proteins deciphered in the stinging ants, venom components of this stingless ant are relatively simple. In addition, the commonly predominant venom components, such as hyaluronidase, metalloproteinase, dipeptidyl peptidase, serine protease, allergen, and neurotoxin, present in stinging ants are not found in the venom of *L. flavus* [4,5,13]. The large diversity of venom compositions yielded by the stinging ants fits to the fact that they suffer from the evolutionary pressure for defending against predators and improved prey capture [4]. But the stingless ants spray the formic acid rich venom for defending and overcome their prey by attacking in large numbers [5]. Thus, they possess fewer proteinaceous venom components over the course of evolution.

Regarding venom phospholipase B identified in *L. flavus*, its transcription was highly detected in venom glands, indicating that it might be an abundant composition. The enzymatic activity of phospholipase B was detected in venoms of snakes, wasps, hornets, and ants (a jumper ant *Myrmecia pilosula*, a bulldog ant *Myrmecia pyriformis,* and a trap-jaw ant *Odontomachus chelifer*) [21,22,23,24,25]. Although phospholipase A2 was commonly found in venoms of stinging ants, phospholipase B has been rarely identified in their venoms [10,13]. Phospholipase B is found in relatively small amounts in a number of snake venoms, which constitutes about 1% of the crude venom [26,27,28]. The venom phospholipase B from snake and wasp has been identified to act as a potent hemolytic agent [27,29].

Odorant binding proteins are small soluble proteins that exhibit the ability to bind, solubilize, and transport hydrophobic odorant molecules and pheromones across the aqueous sensillar lymph to olfactory receptors [30]. Those discovered in chemosensory organs act in chemoreception by detecting molecules present in the environment [31]. They are also expressed in non-chemosensory organs, such as pheromone glands, reproductive organs, and digestive tracts, but their potential functions remain largely uncharacterized [32]. Odorant binding protein has been found in the venom of several parasitoids, bees, and predatory bugs [33,34,35,36,37,38]. An odorant binding protein, AccOBP10, of the Chinese honey bee, *Apis cerana cerana*, with higher transcriptional levels in the venom gland than in other tissues, plays a role in the response to stress conditions [36].

Apolipoprotein D is a lipocalin superfamily member that plays important roles in lipid metabolism, cell differentiation, aging, stress resistance, ootheca formation, and reproduction [39,40,41,42]. Takeout protein has the characteristics of small soluble proteins and is most similar to juvenile hormone binding protein [43]. It is involved in diverse and important processes in insects, such as aging and longevity, insecticide susceptibility, food intake, and courtship [44,45,46]. Carbonic anhydrases are involved in pH regulation, ion transport, metabolic processes, and insecticides resistance [47,48]. In insects, nose resistant to fluoxetine protein has only been characterized in the fruit fly, *Drosophila melanogaster*, which participates in oogenesis and embryogenesis and neurodegeneration [49,50]. Thioredoxin reductase is a selenoprotein that plays important roles in cell proliferation, immunity, response to oxidative stress, lifespan, and development [51,52,53,54,55]. So far, there is no information available for the physiological role of these five proteins as venom components.

Notably, ten of the identified venom compositions of *L. flavus* are novel, most of which are peptidic toxins. This is similar to the venom compositions of several ants where many of them exist with uncharacterized functions, and novel peptidic toxins are commonly found [11,20]. Interestingly, novel venom peptides revealed in the giant red bull ant, *Myrmecia gulosa*, appear to be derived from a single gene superfamily, acting as pain-producing toxins [11]. The evolution and function of the novel venom proteins identified should be further investigated.

## 4. Material and Methods

### 4.1. Ants

Workers (Figure 1A) of *L. flavus* were collected from the wild on the campus of Southwest Forestry University in Kunming, Yunnan, China in August 2021. They were brought to the laboratory. Ants from the same nest were kept together in a small plastic container (15 × 10 × 5 cm) with moistened soil at room temperature. Honey water and first instar cockroaches (*Blatta lateralis*) were provided as food.

### 4.2. Transcriptomic Analysis

The workers of *L. flavus* were anesthetized on ice. Venom glands (Figure 1B) and the carcass (the body without the venom apparatus) were dissected from workers in phosphate-buffered saline (PBS) on an ice plate under a light microscope (Leica MZ 16A, Wetzlar, Germany). About one hundred venom glands were pooled in the same sample. Total RNA was immediately extracted from each sample with TRIzol Reagent (Invitrogen, Carlsbad, CA, USA), according to the manufacture’s protocol. The quantity and quality of total RNA was determined with an Agilent 2100 Bioanalyzer (Agilent technologies, Santa Clara, CA, USA). cDNA libraries were prepared using TruSeq Stranded mRNA Library Prep Kit (Illumina, San Diego, CA, USA). The sequencing was performed by Novogene (Beijing, China) using Novaseq 6000 (Illumina, San Diego, CA, USA) with paired-end reads (150 base pairs). The raw data are available from the NCBI Sequence Read Archive (SRA) with the accession of SRX19009215 and SRX19009216. Raw reads were inspected using FastQC v0.11.9 (https://www.bioinformatics.babraham.ac.uk/projects/fastqc/) and were subjected to eliminate the adapter and low-quality sequences by Trimmomatic v1.4 [56]. Clean data were assembled into transcripts using Trinity v2.8.5 [57]. The redundant transcripts were removed and then clustered into unigenes by Corset v1.09 [58]. The open reading frame (ORF) with a length > 90 bp was predicted and annotated with Transdecoder v5.5.0 [59]. All clean reads were mapped back to the unigenes, followed by calculating the read count and TPM (transcripts-per-million) value that was used to represent the expression levels of corresponding unigenes using the Kallisto Super Wrapper in TBtools v1.0987663 [60]. A differential gene expression analysis was performed using edgeR v3.38.4 with the parameters of log2 fold change ≥ 1.5 (venom gland vs carcass) and padj < 0.05 [61].

### 4.3. Identification of Venom Protein

Based on the transcriptomic data, those genes differentially expressed in venom glands in comparison to the carcass and with high TPM values were screened out, following the method as described by [62]. If the proteins encoded by them have a signal peptide that was predicted with SignalP 6.0 [63], they were assigned as venomous candidates. Then, the proteins encoded by housekeeping genes were removed to obtain the final venom proteome. The amino acid sequences of the identified venom proteins were provided in Table S2. They were annotated by BLASTP v2.10.1 search against the NCBI Nr database.

### 4.4. QPCR

The venom gland, gut, ovary, and carcass (the body without the venom apparatus, gut, and ovary) were dissected from workers of *L. flavus*. Samples of each tissue from at least 20 workers were pooled as one biological replicate. Three biological replicates of each tissue were analyzed in this study. Total RNA was isolated using TRIzol reagent (Invitrogen, Carlsbad, CA, USA), according to the manufacture’s protocol. After being quality accessed with the 1% agarose gel electrophoresis, 1 µg total RNA was used to synthesize the cDNA with the PrimeScript RT Reagent Kit with gDNA Eraser (TaKaRa, Dalian, China). The GAPDH (glyceraldehyde-3-phosphate dehydrogenase) gene was used as an internal reference gene. Gene sequences of venom proteins were retrieved from the transcriptomic data, as described above. Gene specific primers were designed using Primer Premier 6.0 (PREMIER Biosoft International, Palo Alto, CA, USA) (Table S2). qPCR was performed on a qTOWER 2.2 Real Time qPCR Thermal Cycler (Analytik Jena AG, Jena, Germany) with Bestar® SybrGreen qPCR mastermix (DBI^®^ Bioscience, Shanghai, China). qPCR conditions were as follows: initial denaturing at 95 °C for 2 min, followed by 40 cycles, each comprising 95 °C for 10 s of denaturing, 58 °C for 31 s of annealing, and extension for 30 s at 72 °C. A melting curve analysis was performed from 60 °C to 95 °C to determine the specificity of qPCR primers. The Q-gene method was applied to process the qPCR data [64,65]. Gene expression data were statistically analyzed using GraphPad Prism 8.0 (GraphPad Software Inc., San Diego, CA, USA) with one way analysis of variances (ANOVA) (*p* < 0.05) and visualized with GraphPad Prism 8.0 (GraphPad Software Inc., San Diego, CA, USA).

## Figures and Tables

**Table 1:** Overview of *Lasius flavus* transcriptome.

Total raw reads from venom gland	41,335,556
Total raw reads from carcass	21,846,927
Total clean reads from venom gland	40,450,976
Total clean reads from carcass	21,100,589
Average Q20 (%)	97.62
Average Q30 (%)	92.65
Total number of transcripts	164,806
Total number of unigenes	51,015
Average length of transcripts (bp)	1083.76
Average length of unigenes (bp)	895.17
N50 length of transcripts (bp)	219
N50 length of unigenes (bp)	263

**Table 2:** Venom proteins of *Lasius flavus* discovered by transcriptomic approach.

Protein Name	Read Count-Vg	Read Count-Ca	Log2 Fold Change (Vg/Ca)	*p* Value
Phospholipase B	25,763	7835	3.70	6.44 × 10^−5^
Odorant binding protein	10,438	5572	2.88	1.24 × 10^−3^
Apolipoprotein D	34,783	38,031	1.85	3.13 × 10^−2^
Takeout protein	32,836	718	7.49	1.01 × 10^−11^
Carbonic anhydrase	2062	1797	2.18	1.23 × 10^−2^
Nose resistant to fluoxetine protein	57,637	1774	7.00	8.12 × 10^−11^
Thioredoxin reductase	4508	1882	3.24	3.54 × 10^−4^
UN1	30,970	4755	4.68	1.32 × 10^−6^
UN2	4466	1997	3.14	5.06 × 10^−4^
UN3	5387	227	6.55	6.53 × 10^−10^
UN4	16,412	1944	5.06	2.87 × 10^−7^
UN5	3539	115	6.92	1.47 × 10^−10^
UN6	5728	1493	3.92	2.79 × 10^−5^
UN7	41	0	9.66	1.21 × 10^−6^
UN8	7539	225	7.04	7.84 × 10^−11^
UN9	754	317	3.23	4.00 × 10^−4^
UN10	5213	2998	2.78	1.79 × 10^−3^

Vg, venom gland; Ca, carcass (body deprived of venom apparatus).

## Data Availability

The datasets used and analyzed during the current study are available from the corresponding author on reasonable request.

## Supplementary Materials

The following are available online at https://www.mdpi.com/article/10.3390/toxins15020106/s1, Figure S1. Top BLAST hit species distribution of the *Lasius flavus* transcriptome assembly, Figure S2. GO annotation of the unigenes, Table S1. Amino acid sequences of venom proteins identified from *Lasius flavus*, Table S2. Gene-specific primers used for quantitative real-time PCR.
