# Supplementary figures and images for: Proteinaceous Venom Expression of the Yellow Meadow Ant, Lasius flavus (Hymenoptera: Formicidae)
Source: Toxins (Basel). 2023 Jan 26;15(2):106. doi: 10.3390/toxins15020106 (PMC9961005; doi:10.3390/toxins15020106)

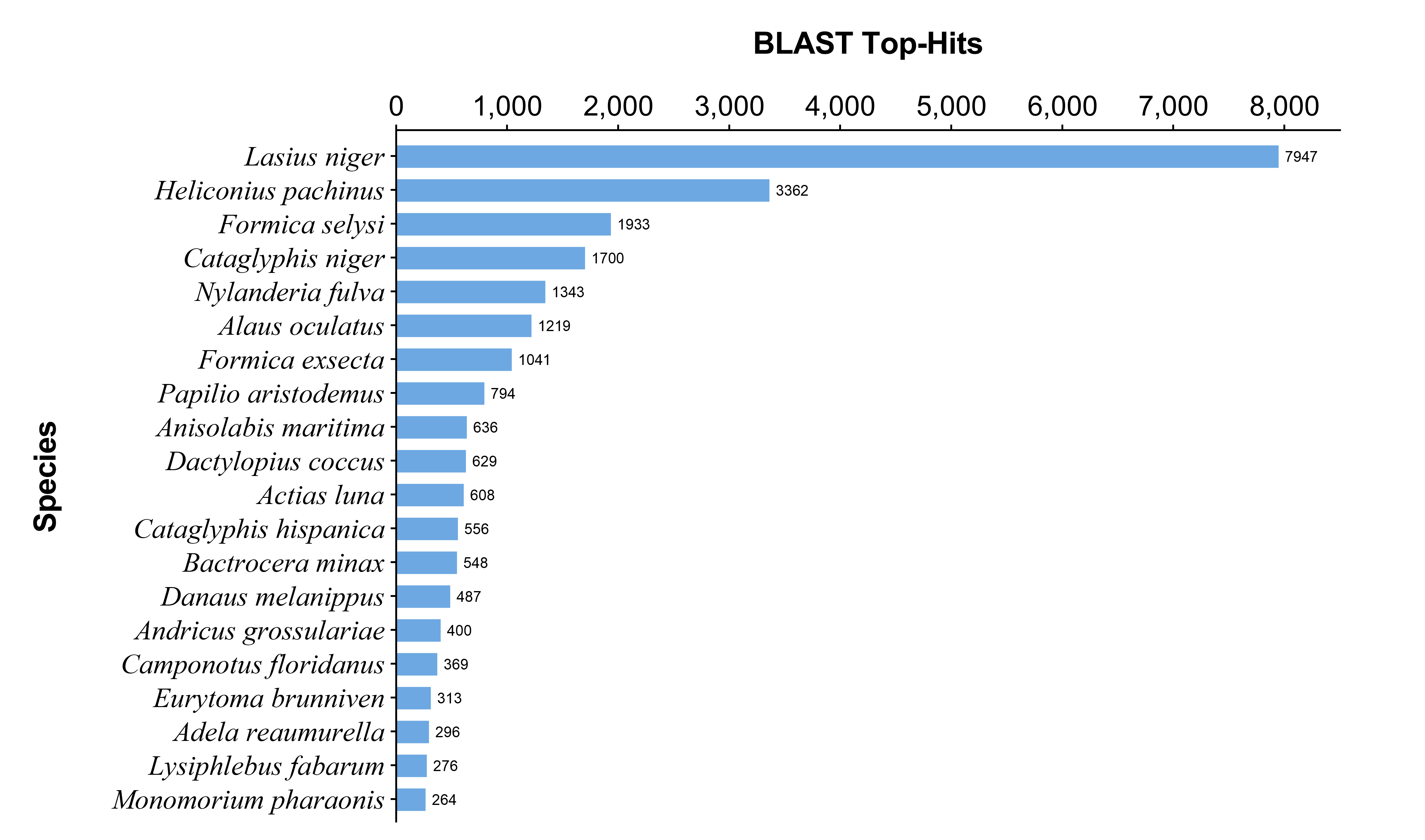

Supplement: Supplementary file 1 [file toxins-15-00106-s001.zip › Figure S1.tif]

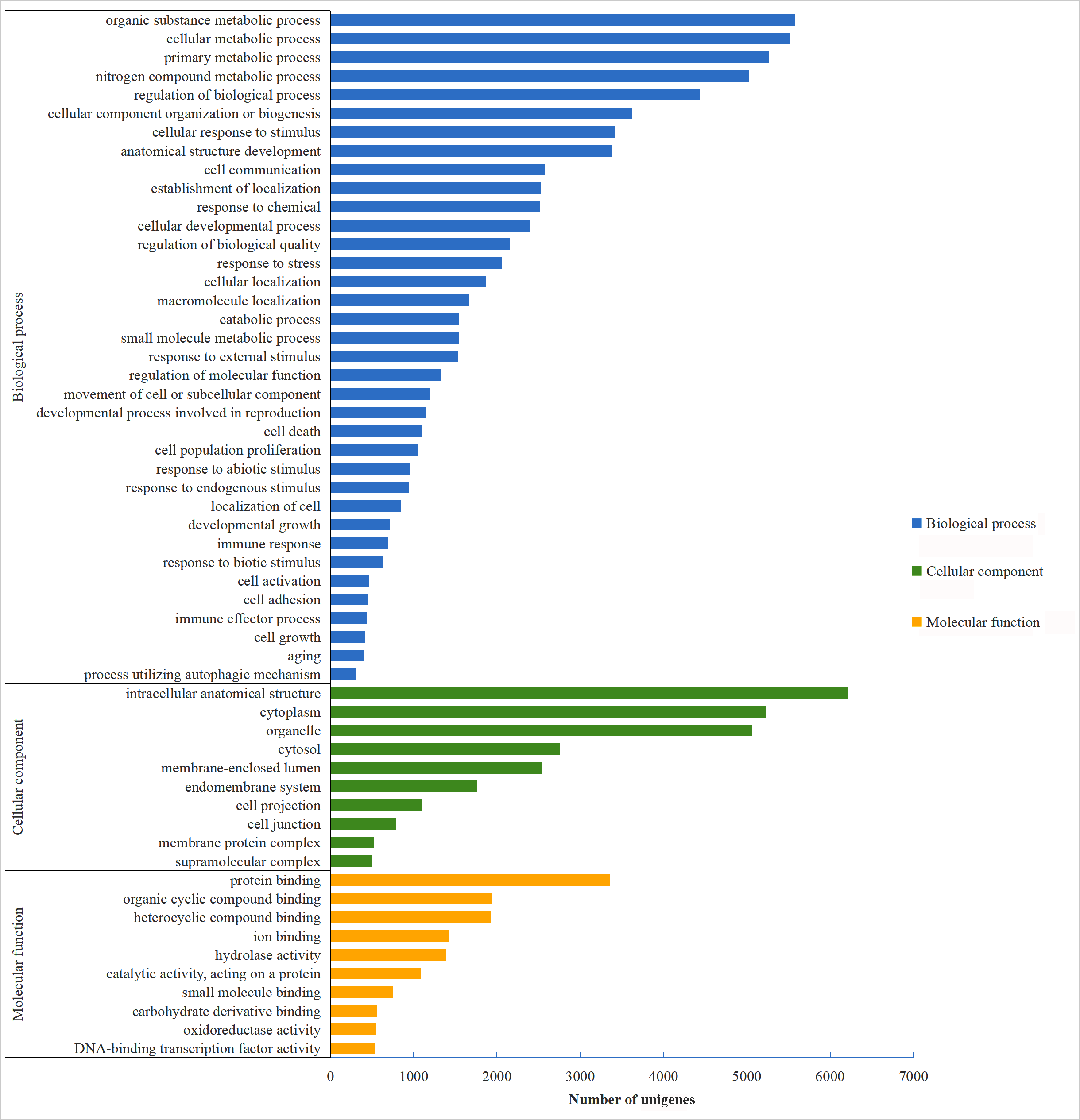

Supplement: Supplementary file 1 [file toxins-15-00106-s001.zip › Figure S2.tif]
